# A multistep deep learning framework for the automated detection and segmentation of astrocytes in fluorescent images of brain tissue

**DOI:** 10.1038/s41598-020-61953-9

**Published:** 2020-03-20

**Authors:** Cihan Bilge Kayasandik, Wenjuan Ru, Demetrio Labate

**Affiliations:** 1 Istanbul Medipol University, Department of Computer Engineering, Istanbul, Turkey; 2 Center for Neurodegeneration, Houston Methodist Research Institute, Houston, Texas United States of America; 3 University of Houston, Department of Mathematics, Houston, Texas United States of America

**Keywords:** Neuroscience, Mathematics and computing

## Abstract

While astrocytes have been traditionally described as passive supportive cells, studies during the last decade have shown they are active players in many aspects of CNS physiology and function both in normal and disease states. However, the precise mechanisms regulating astrocytes function and interactions within the CNS are still poorly understood. This knowledge gap is due in large part to the limitations of current image analysis tools that cannot process astrocyte images efficiently and to the lack of methods capable of quantifying their complex morphological characteristics. To provide an unbiased and accurate framework for the quantitative analysis of fluorescent images of astrocytes, we introduce a new automated image processing pipeline whose main novelties include an innovative module for cell detection based on multiscale directional filters and a segmentation routine that leverages deep learning and sparse representations to reduce the need of training data and improve performance. Extensive numerical tests show that our method performs very competitively with respect to state-of-the-art methods also in challenging images where astrocytes are clustered together. Our code is released open source and freely available to the scientific community.

## Introduction

The human brain is a complex network of over 2 × 10^11^ neural cells comprising neurons and glial cells. Neuroglia comprise astrocytes, oligodendrocytes, NG2 glia, microglia and all peripheral glia^1^. Astrocytes, a subtype of glial cells with a complex star-shaped morphology, are the most abundant cells in most part of the human brain^2^. Although they were long characterized as supportive cells only involved in maintaining neuron homeostasis and survival, a number of studies during the last decade have revealed that astrocytes play an active role in fundamental brain processes underlying neuronal development and function. Several studies have implicated astrocytes in controlling the development of the nervous system through axon guidance and synaptogenesis. During neural circuit development, astrocytes are responsible for the regularization of the neuronal network by pruning abnormal or dysfunctioning synapses^3^. In addition, together with the pre- and post-synaptic parts of two neuronal synapses, astrocytes can form a so-called “tripartite” synapse that helps modulate synaptic transmission via the release of neurotransmitters such as glutamate and ATP^4,5^. The critical roles of astrocytes in neuronal development and connectivity make them among the most promising targets for innovative therapies designed to treat a range of brain disorders or neurological injuries^6^. As a result, the attention on astrocytes has dramatically increased in recent years.

Astrocytes have been shown to reflect their diverse abilities and functions on their special structural design, and alterations in astrocyte morphology are known to correlate to traumatic brain injury, infection, ischemia, autoimmune responses, and neurodegenerative diseases^5,7,8^. For instance, their intricate arborization and ramifications allow them to enwrap synaptic terminals and modulate synaptic processes. Additionally, astrocytes have the ability to swell and shrink in size – a property that allows them to reduce the leakage of neurotransmitters increasing the active concentration in the synapse, and preventing spillover in the case of damage. The functional relevance of morphological changes in astrocytes is further supported by studies relating cell morphology to the expression level of markers of astrocyte activation^3,8,9^.

In spite of the effort to advance understanding of structure-function properties of astrocytes, though, progress in this field has been slower due to the difficulty in adapting conventional algorithms for image analysis and cell segmentation to images of astrocytes and other glial cells. Automated analysis of microscopy images of astrocytes is particularly challenging due to their large variability in size and shapes, the complex topology of processes occurring over multiple scales and the highly entangled nature of networks formed by such cells.

There are currently a relatively small number of image analysis methods targeted to glial cells, including astrocytes. The simplest methods adapt classical intensity thresholding techniques. Healy *et al*.^10,11^ reviewed several automated thresholding strategies available as plugins in FIJI and found that the performance of threshold-based segmentation of fluorescent images of astrocytes and other glial cells “varies considerably in quality, specificity, accuracy and sensitivity with entropy-based thresholds scoring highest for fluorescent staining”. The main drawback of such methods is that their performance is inconsistent and often unpredictable as it depends on a multiplicity of factors including conditions of acquisition, cell density and noise level, so that a laborious manual tuning of parameters is required to guarantee good performance. Another critical drawback is that such methods are unable to automatically separate entangled cells in the image. To address such challenge, Suwannatat *et al*.^12^ have proposed a probabilistic method that uses random walks to separate cells^13^. This method, however, uses a manual approach to detect each cells and its performance degrades rapidly if more than two cells are entangled. Another approach, proposed by Kulkarni *et al*.^14^, identifies astrocytes in fluorescent images using a fluorescent marker of the nucleus (DAPI), and then traces the cell arbors using a tracing method called *local priority based parallel (LPP) tracing*. Authors show that reconstructed traces are useful to define quantitative arbor measurements via Scorcioni’s L-measures^15^, a rich collection of neuroanatomical parameters for the quantitative characterization of neuronal morphology. However this method does not generate voxel-level segmentation and hence does not address the problem of cell separation. More recently, Yang *et al*.^16^, have introduced a method for the analysis of images of astrocytes in 3D resolution that applies a convolutional neural network (CNN) to segment 3D images in combination with a shape prior computed by a multiplicative Voronoi diagram to separate touching cells. Finally, the recent work by Suleymanova *et al*.^17^ introduces a method for astrocyte detection based on a CNN that provides very accurate result even though training requires a large number of training samples. However, this method does not address the problem of segmentation.

In this paper, we introduce an innovative image analysis framework for automated detection and segmentation of astrocytes in 2-dimensional fluorescent images. Our approach brings some significant novel contributions. First, we introduce an innovative method for the automated detection of astrocytes in fluorescent images based on multiscale directional filters that does not require any nucleus marker. A major advantage of this approach is the ability to accurately detect and separate cells that are very close together or even contiguous. Another main novelty is that we introduce a modified CNN architecture for astrocyte segmentation that facilitates training and reduces the need of training samples by imposing a geometric bias on the convolutional filters. We validate our algorithm on fluorescent images of astrocytes from different brain regions, including images where astrocytes are densely packed and clustered together. We show that our algorithm is extremely competitive with state-of-the-art methods in terms of accuracy and computational efficiency.

By providing an accurate and automated framework for the detection and segmentation of astrocytes, our method also provides a platform to facilitate quantitative analysis of astrocytes through the extraction of morphological features that could be used to classify astrocyte sub-populations or to establish phenotypic characteristics.

## Materials and Methods

### Method

**Figure 1:**
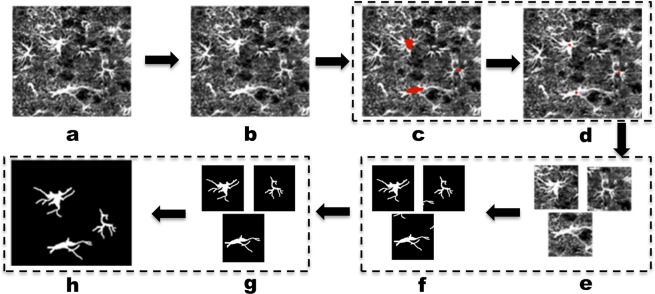
Workflow of astrocyte detection and segmentation pipeline. Input fluorescent image (**a**) is first preprocessed to remove noise (**b**); next cells are detected using the Directional Ratio method (**c**) and locating the cell centroids (**d**); after identifying a region of interest containing a single cell (**e**), each cell is segmented (**f**) using our CNN; finally, segmented images are postprocessed to remove unconnected components and discard objects that are not recognized as astrocytes (**g**); next they are refitted into original location of the input image (**h**).

As summarized above, methods based on intensity thresholding for cell segmentation are general-purpose and simple to implement. However, they typically require a significant manual intervention to achieve a satisfactory performance. Machine learning methods on the other hand are more complex and require a training step, but have generally proven to achieve superior performance in applications from biomedical imaging. Processing images of astrocytes though is especially challenging due to the highly complex cell morphology and the need to separate entangled cells. To address this task, we introduce a novel multi-step algorithmic pipeline that first detects each astrocyte in the image (without assuming any cell marker) and next segments each cell separately. The complete pipeline is illustrated in Fig. 1 and consists of the following steps:

1. *Pre-processing*. It removes image noise to facilitate the successive processing steps.
2. *Cell detection*. It finds regions of interest associated with a single astrocyte.
3. *Cell segmentation*. It separates the astrocyte from the background.
4. *Post-processing*. It refines the segmentation process by eliminating objects that do not satisfy prescribed shape criteria.

We describe below how we implemented each processing step by devoting special attention to cell detection and cell segmentation - our main novel contributions in the algorithm.

#### Data pre-processing

We pre-process images acquired through confocal microscopy to remedy possible degradation introduced during the acquisition process and facilitate the successive processing steps of the algorithm. The main source of degradation is the Poisson noise introduced by the photon-counting process at the detector. To remove noise, we applied a denoising algorithm based on shearlets and adaptive thresholding developed by one of the authors^18^. Unlike more traditional denoising methods, this approach is especially effective at preserving cell boundaries due to the high directional sensitivity of shearlet filters^19,20^. Images were also normalized with respect to pixel intensity.

#### Cell detection

Astrocytes are visible in the channel marked by GFAP (Glial fibrillary acidic protein), a marker used to distinguish astrocytes from other glial cells. Methods for cell detection relying on contrast enhancement and intensity thresholding (cf.^10,11^) are the simplest to implement. However they require a manual intervention to perform competitively and are unable to separate contiguous cells - a common situation in fluorescent images of brain tissue where astrocyte form dense networks. Alternatively, one can apply DAPI, a nucleus marker, to the image. However, this marker is not astrocyte-specific and, since any image will typically contain DAPI-marked locations associated with other cell types, the marker cannot be used directly to detect astrocytes^14^. To avoid this issue, in this paper we apply a new approach to automatically detect astrocytes based on the *Directional Ratio*, a multiscale geometric descriptor that estimates the probable location of cell bodies in an image by measuring local anisotropy over multiple scales^21,22^. This method is purely morphology-based, does not require any nucleus marker and was originally developed by the authors to detect somas in fluorescent images of neurons^23^.

### Definition

*Given a collection of multiscale orientable filters*  *ϕ*_*j*,*ℓ*_, *where the indices j and*  *ℓ*  *are associated with a range of scales and orientations, respectively, the (modified) Directional Ratio of an image f at the j-th scale and at location p is the quantity*

1 $${D}_{j}f(p)=\frac{{\min }_{\ell }| \,f\ast {\phi }_{j,\ell }(p){| }^{2}}{{\max }_{\ell }| \,f\ast {\phi }_{j,\ell }(p)| }.$$

In a nutshell, *D*_*j*_ *f*(*p*) computes the ratio of the smallest directional filter response at location *p* and scale *j* over the largest one. Hence its value ranges between 1 if *p* lies in a locally isotropic region (filter response is not directional-sensitive) and 0 if *p* lies withing a local vessel-like structure (filter response is very dependent on the orientation). The formula above is a slight modification of the original one, where power-2 in the numerator is useful to enhance the filter response in images with relatively low contrast.

By computing the Directional Ratio of an image and applying a threshold (we used 0.7 for our images), we distinguish locally isotropic regions – those corresponding to potential cell body locations – from the rest of the image. As shown in the Results section below, this method can reliably identify a region inside the cell body of an astrocyte despite its highly irregular shape. By computing the centroid of such region and selecting a rectangular window of appropriate size (128 × 128 pixels in our Results) centered at this location, we then extract a rectangular patch containing a single astrocyte plus possible processes from contiguous cells. Any such patch is then fed into the segmentation routine below.

#### Cell segmentation

**Figure 2:**
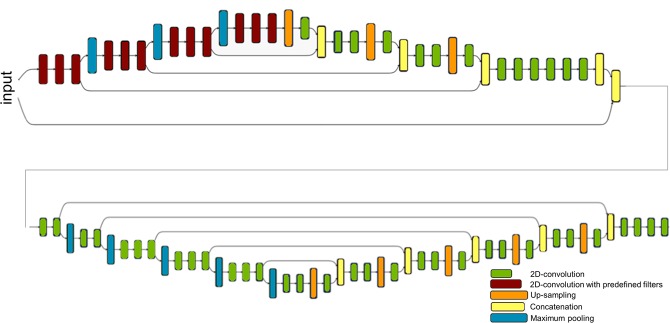
The proposed Geometric-Enhanced Stacked U-net architecture. Our GESU-net consists of two stacked U-nets. The first U-net includes filters (black boxes) in the encoding sections that are selected from a family of multiscale directional filters. The second U-net is fed by concatenating its input to the output of the first U-net. The weights of the encoding section of the second U-net are transferred from a VGG-16 network pretrained on Imagenet.

Our segmentation method is based on a new CNN that leverages a geometric-enhanced enconder-decoder network and transfer learning. The proposed network architecture is illustrated in Fig. 2 and consists of two consecutive modified U-nets where the first U-net learns a cell representation and the second one generates a segmentation map. We call this network Geometric-Enhanced Stacked U-net (GESU-net).

Recall that a CNN is a neural network consisting of a sequence of convolutional and pooling layers, followed by a fully connected layer^24^. In a CNN, the first section performs feature detection on the input data while the last section performs a classification on top of the extracted features. A U-net^25^ is special CNN where the feature-extracting (encoding) section of the network is followed by a decoding section where pooling operations are replaced by upsampling operators. As a result, the expansive section is approximately symmetric to the contracting section, yielding a u-shaped architecture. U-nets have been shown to perform successfully in problems of biomedical image segmentation.

A major novelty of our GESU-net is that the encoding section of the first U-net is modified by imposing that its convolutional filters are selected from a class of predefined shearlet-like filters. This class of filters consists of a low-pass and of multiple high-pass directional filters designed to be both highly sparse and directional sensitive according to the framework of multiscale directional framelets in^26^. During training, only the coefficients of the linear combination are learned rather than the entire filter weights. By reducing the flexibility of each convolutional filter while imposing a geometric constraint, this approach has the advantage of facilitating the training step. This method is inspired by the theory of structured receptive fields^27^ where this idea was originally proposed in order to reduce the number of network parameters in problems with small data size.

The output of this first U-net is concatenated with the input image and then fed into a second modified U-net architecture. Here the contracting part of the network uses weights that are transferred from a VGG16 network trained on ImageNet with the goal to improve the efficiency of the representation^28^.

##### Justification of proposed of GESU-net

To motivate and justify the proposed GESU-net, we compared its performance against alternative simpler architectures consisting of single U-nets.

**Figure 3:**
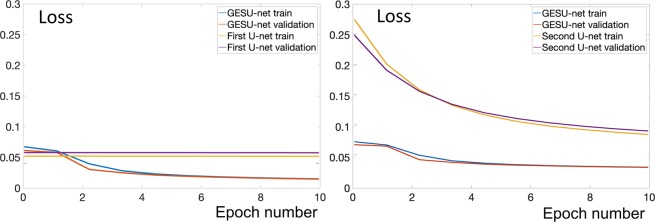
Advantages of transfer weights and pre-defined filters: *Left:* When transfer learning section is eliminated from the GESU-net and only the first U-net with pre-defined filters is used, the capability of the model decreased significantly. Learning is slower and loss converges to a higher value. *Right:* When pre-defined filters are eliminated and only the second U-net with transferred weights is used, training starts with a large error and loss value converges more slowly.

**Figure 4:**
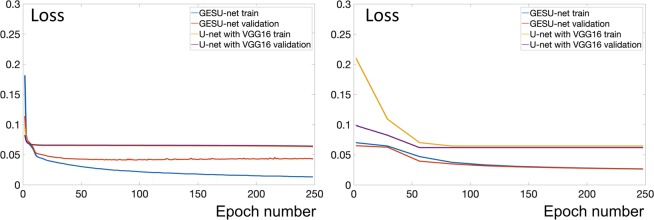
Benefits of including predefined filters for different training dataset sizes: *Left:* Loss function where %75 of training samples are used to train the models; no data augmentation. *Right:* Loss function using entire train dataset and data augmentation.

We first examined a simplified network obtained by removing the second U-net from our GESU-net. Hence the resulting network is a modified U-net whose encoding section learns linear combinations of predefined filters according to the strategy described above. As shown in Fig. 3 (left), the learning capability of this simpler network decreases with respect to the GESU-net. The loss function decays more slowly and it converges to a higher error value indicating that the modified U-net with transferred weights which is part of the GESU-net is important to improve segmentation accuracy. We next examined another simplified network obtained by eliminating the first U-net from our GESU-net; that is, the simplified network is a U-net with transferred weights in the contracting section. Fig. 3 (right) shows that the loss function decreases very slowly as compared with the GESU-net. Fig. 4 shows that the slow convergence of the loss function and the inferior performance is especially evident when the size of training data size is small showing the importance of predefined filters to facilitate training.

We remark, that even though the difference in loss values shown in Figs. 3 and 4 is apparently small, this can lead to a significant difference in the segmentation quality. For an image of size 1024 × 1024, a %2 increase in loss value corresponds to over 300 miss-classified pixels and this can cause a significant error since the average area of an astrocyte is about 1500 pixels.

To quantitatively assess the impact of each block of our network into the overall design, in the Results section we compare the performance of our GESU-net against a simple U-net and a U-net with a pre-trained VGG16 module.

##### Network training and parameter setting

Our GESU-net was implemented in Python 2.7+ using the standard deep learning library Keras^29^. Our code is available in https://github.com/cihanbilge/AstrocyteSegmentation. All our computations were carried out using *Intel Xeon CPU E5-2699 v4 @ 2.20 GHz–32 CPUs 256 GByte RAM 300 GByte IBM Storwize V7000 Flash Drive*.

To train our network for segmentation, we extracted patches from three images (fluorescent images of mice cortex) of size 1024 × 1024. Specifically, after preprocessing the images to remove noise, we applied our cell detection routine to generate sub-images of size 128 × 128 pixels containing single astrocytes; next, astrocytes in the sub-images were manually segmented by domain experts. Through this procedure we generated 118 training images of size 128 × 128 containing manually segmented astrocytes. As the size of training samples is small and it is impractical to increase it due to the laborious annotation process (and limited availability of experimental data), we applied classical data augmentation to increase the training size^30^. Specifically, we generated 20 extra images for each training image by applying rigid translations and rotations.

After training, we found that the mean absolute error of the loss function was 0.0004 for the training data and 0.0007 for the validation data.

#### Post-processing

**Figure 5:**
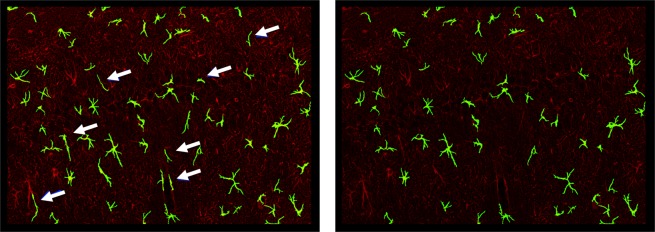
Post-processing eliminates string-like objects. *Left:* Segmentation before post-processing. *Right:* Segmentation after post-processing. Arrows point to objects being eliminated.

Before re-assembling the segmented image patches into the final segmented image, we need to (i) eliminate possible processes from neighboring cells contained in the patch; (ii) discard segmented cells that do not satisfy prescribed shape criteria and are not recognized as mature astrocytes. The first task is due to the fact that, during the process of generating a patch centered on a detected cell, it is possible that processes from neighboring cells are included in the patch. We eliminate these processes by the segmented patches using standard method to identify and then discard disconnected image components that are below a certain size (see Fig. 1, steps f-g). The second task is due to the presence in our images of string-like GFAP-stained objects that can be associated with astrocytes precursors (non-mature cells that have not yet acquired a stellate morphology^31^). To eliminate these objects, we developed a method to separate star-shaped objects from string-like ones that applies the same type of directional filters used during cell detection. In brief, we applied our directional filters (with 20 orientations) and tested for prominent directional responses. If a shape is star-like, filtering generates at least 2 prominent directional responses due to the presence of processes with at least 2 distinct orientations. If a shape is string-like, filtering generates one prominent directional response only. Figure 5 shows that using this method we can detect and eliminate objects that do not satisfy this criterion for star-shape morphology. In the Result section, we include numerical tests to quantify the effect of our post-processing module on the overall performance.

### Preparation of samples and image sets

For the development of our algorithm and part of our numerical validation, we used fluorescent microscopy images of mouse brain previously collected^32^ in the laboratory of Dr. S. Tang from the Department of Neuroscience, Cell Biology and Anatomy at the University of Texas Medical Branch.

#### Animals

Animal procedures and experiments were in line with standards and regulations to reduce the suffering of the animals approved by the Institutional Animal Care and Use Committee (IACUC) of University of Texas Medical Branch (UTMB). UTMB complies with the USDA Animal Welfare Act (Public Law 89-544) as amended by PL91-579 (1970), PL94-279 (1976) and 45 CFR37618 (1980); Health Research Extension Act of 1985 (Public Law 99-158); follows the Public Health Service Policy on Humane Care and Use of Laboratory Animals (revised September, 1986); and the Guide for the Care and Use of Laboratory Animals (revised September 1986); the Guide for the Care and Use of Laboratory Animals DHEW (NIH) 85–23 revised 1985.

#### Immunohistochemistry

Briefly, mice were anesthetized with inhaled isoflurane and perfused transcardially with PBS. Brains were isolated rapidly. Tissues were fixed in 4% paraformaldehyde in 0.1 M PBS overnight, cryoprotected in 30% sucrose for at least 24 hrs at 4 °C, and then embedded in optimal cutting temperature (OCT) compound (Sakura Finetek, Torrance, CA). Frozen sections (35 *μ*m) were prepared for immunofluorescent staining.

For immunofluorescent staining, sections were rinsed with PBS two times and incubated with blocking buffer (5% with 0.3% Triton X-100 in PBS) for 2 hrs at room temperature (RT). After incubation with with anti-GFAP (1:500) in blocking buffer for 24 hrs at at 4 °C. Then, the sections were incubated with secondary antibody for 2 hrs at RT and stained with DAPI to visualized nuclei before mounting.

All images were captured using a confocal microscope system by Nikon. 15–25 consecutive optical sections with 1 *μ*m interval thickness at 20x magnification were captured for each z-stack image. Acquisition parameters, including photomultiplier gain and offset, were kept constant throughout each set of experiments.

### Evaluation metrics

To assess detection and segmentation performance, we used the standard binary classification metrics of sensitivity, precision and Dice’s coefficient^33^.

The *sensitivity* (or True Positive Rate or *recall*) measures the proportion of correctly detected cells (or points) with respect to the total number of cells that are manually identified by a domain-expert without knowledge of the algorithm results. Denoting by TP (=true positive) the number of correct detections and by FN (=false negative) the number of missed detections, we define the sensitivity as

2 $${\rm{S}}=\frac{TP}{TP+FN}$$

The *precision* measures the proportion of correctly detected cells over all detected cells. That is, denoting by FP (=false positive) the number of wrongly detected cells, the precision is

3 $${\rm{P}}=\frac{TP}{TP+FP}$$

Finally, the *Dice’s Coefficient* (also known as *F1 score*) can be considered a measure of the overall effectiveness of the classification algorithm and is given by

4 $${\rm{DC}}=\frac{2TP}{2TP+FP+FN}.$$

DC ranges between 0 and 1 with *DC* = 1 describing perfect classification.

## Results

In this section, we illustrate the application of our method for the detection and segmentation of astrocytes. As a validation set, we used a collection of 5 GFAP-stained fluorescent images of mice astrocyte cells in the cortex collected by the laboratory of Dr. Tang from the Department of Neuroscience and Cell Biology at the University of Texas Medical Branch. We refer below to this set as Set_1. To further validate the performance of our method, we also considered 11 randomly selected images from the publicly available image set BBBC042 from the Broad Bioimage Benchmark Collection (https://data.broadinstitute.org/bbbc/image_sets.html)^34^. This set consists of brightfield images of rat astrocyte cells in different brain regions containing chromogen-based staining against GFAP and will be henceforth denoted as Set_2. Both image sets Set_1 and Set_2 were acquired using 20x magnification. Images in Set_1 were manually labeled (full cell segmentation) by domain experts without prior knowledge of our results; annotations (detection only) for the images in Set_2 were also generated by domainn experts and are provided in the website indicated above.

### Astrocytes detection

**Figure 6:**
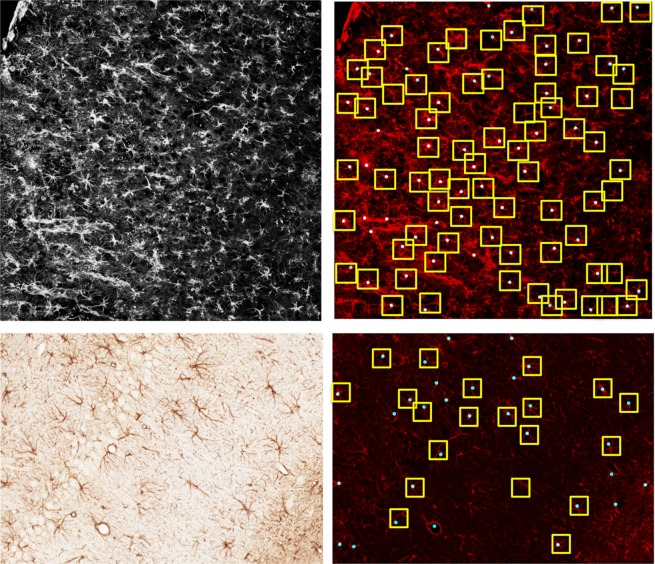
Astrocyte detection performance. *Left:* Representative images from set Set_1 (top) and Set_2 (bottom). *Right:* Detected astrocytes are tagged with blue dots. The yellow boxes contain the astrocytes that were manually detected by domain experts.

We start by assessing the astrocyte detection performance. Figure 6 illustrates the astrocytes detection performance of our method on representative images from the image sets Set_1 and Set_2. Images in Set_1 exhibit a dense astrocyte population of about 30-65 cells per image. Images in Set_2 are less dense and contain about 10-30 cells per image. The detection results show that our method is very effective also in the situation of cells that are relatively close together. However, images in Set_1 occasionally contain small sub-regions of highly dense clusters of astrocytes where even manual annotators were unable to separate cell boundaries reliably. Since it is not possible to evaluate the performance of our method in these (small) regions, we ignored these regions in our analysis.

To quantify performance of our algorithm for astrocytes detection, we examined its performance on the 5 images in Set_1 and the 11 images we extracted from Set_2. We remark that the two image sets are comparable with respect to cell count since Set_1 contains a total of 231 astrocytes and Set_2 contains a total of 227 astrocytes. We found that our method correctly detected 404 cells out of 458 cells. To benchmark our detection performance against existing and state-of-the-art methods, we applied on the same dataset the ILASTIK software^35^ – a general purpose cell detection and classification software –, the algorithm FindMyCells (FMC)^17^ – a recent cell detection software especially targeted to astrocytes, and a standard U-net. All these methods rely on a supervised learning approach with the difference that while FMC and U-net use a deep learning approach, ILASTIK uses traditional machine learning (random forest).

We report detection performance results for each method in Table 1, where we list the performance metrics of sensitivity (S), precision (P) and Dice’s coefficients (DC). The table shows that our method performs very competitively overall. The FMC algorithm performs slightly better that our algorithm on Set_2 – the set on which the FMC algorithm was developed – but significantly worse than our method on Set_1. The slightly lower performance of our method on Set_2 is due to the more elongated shape of astrocytes in Set_2 (astrocyte morphology is region-dependent) with respect to Set_1 that was used to develop our method. Despite this difference though, our method’s performance is very satisfactory. ILASTIK performs rather poorly on these image sets due to the known difficulty of detecting astrocytes as compared to cells of more regular shape (see additional comments in the Discussion below). The performance of the standard U-net is, as expected, intermediate between our GESU-net and FMC: its DC value on Set_1 is better than FMC but worse than GSU-net and, on Set_2, its DC value is worse than FMC and worse than GESU-net.

**Table 1:** Astrocytes detection performance using our GESU-net algorithm, a standard U-net, FindMyCells (FMC)^17^ and ILASTIK^35^.

Dataset	GESU-net			U-net			FMC			ILASTIK		
	S	P	DC	S	P	DC	S	P	DC	S	P	DC
Set_1 (231 cells)	0.90	0.72	0.80	0.92	0.57	0.71	0.30	0.71	0.42	0.40	0.14	0.21
Set_2 (227 cells)	0.87	0.59	0.70	0.96	0.21	0.35	0.68	0.83	0.75	0.56	0.18	0.28

Performance metrics include Sensitivity (S), Precision (P) and Dice’s Coefficient (DC).

### Astrocytes segmentation

We next examine the task of astrocyte segmentation. As we observed above, the major challenge in address such task is the accurate recovery of the finer processes of each cell as they are often difficult to tell apart from the background. Another challenge is the separation of cells that are close together and have overlapping processes.

**Figure 7:**
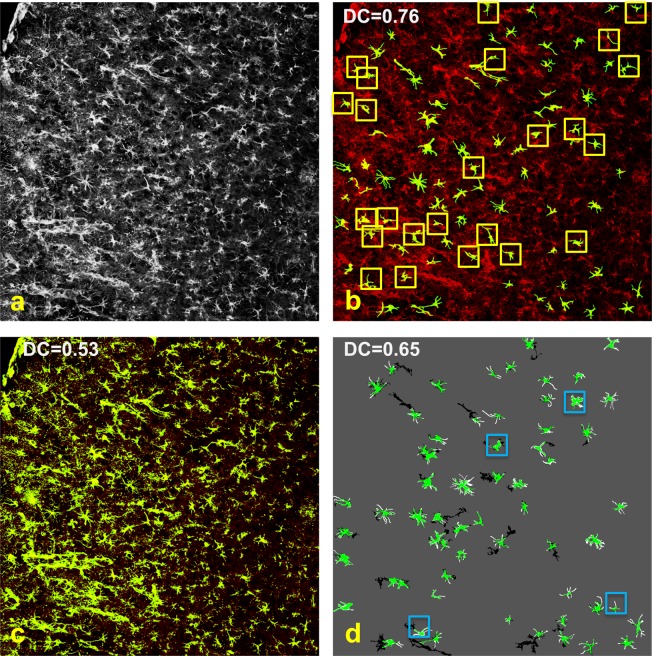
Astrocyte segmentation. (**a**) GFAP-stained astrocyte image of mouse cortex displayed in gray-scale and (**b**) corresponding segmented astrocytes generated by our GBSU-net (green) overlaid on the original image (red). (**c**) Segmentation of the same image generated by the BioVoxxel plugin in FIJI^11^ and (**d**) segmentation generated by applying the BioVoxxel plugin locally to the astrocytes detected by our method (color green: True Positive; black: False Positive; white: False Negatives). Yellow boxes in (**b**) contain cells detected by GESU-net but not by modified BioVoxxel in (**d**) and, vice versa, blue boxes in (**d**) contain cells detected by modified BioVoxxel but not by GESU-net (**b**). Dice’s Coefficient (DC) is indicated on top with additional measures reported in Table 2.

 Figure 7 illustrates the segmentation performance of our method on a representative image from Set_1 that contains 65 astrocytes (according to manual annotation). As shown by the figure, our method is very efficient to recover the cell bodies of most astrocytes and the processes emanating from it. We report the corresponding quantitative performance results in Table 2 using again Sensitivity, Precision and Dice’s Coefficient. Note that, to compute these quantities in this case, TP is the number of correctly identified pixels, FN the number of missed pixels and FP the number of detected cells that are wrongly identified.

**Table 2:** Astrocyte segmentation performance on a representative image from our dataset Set_1 (same as Fig. 7) measured using Sensitivity (S), Precision (P) and Dice’s Coefficient (DC).

	S	P	DC
GESU-net	0.69	0.86	0.76
U-net	0.77	0.55	0.64
U-net with VGG16	0.87	0.58	0.70
BioVoxxel	0.62	0.46	0.53
BioVoxxel with GESU-net detection	0.69	0.61	0.65

The performance of our GESU-net algorithm is compared against a standard U-net, a U-net with pretrained VGG16 module, the BioVoxxel toolbox in FIJI^11^ and a local application of the same toolbox on the cells detected by our method based on Directional Ratio.

To benchmark our result, we compared our method against the BioVoxxel plugin in FIJI^11^ – an automated method for astrocyte segmentation that uses intensity thresholding – and against a standard U-net^25^. The visual comparison of the BioVoxxel segmentation in Fig. 7(c) with our result shows that this method performs rather poorly as it misses many cell processes and is unable to separate close or contiguous cells. The quantitative result in Table 2 underscores the poor performance of BioVoxxel on our data (DC = 0.53 vs DC = 0.76 for our method). The U-net performs significantly better (DC = 0.64) than BioVoxxel but worse than our more sophisticated deep learning strategy.

**Figure 8:**
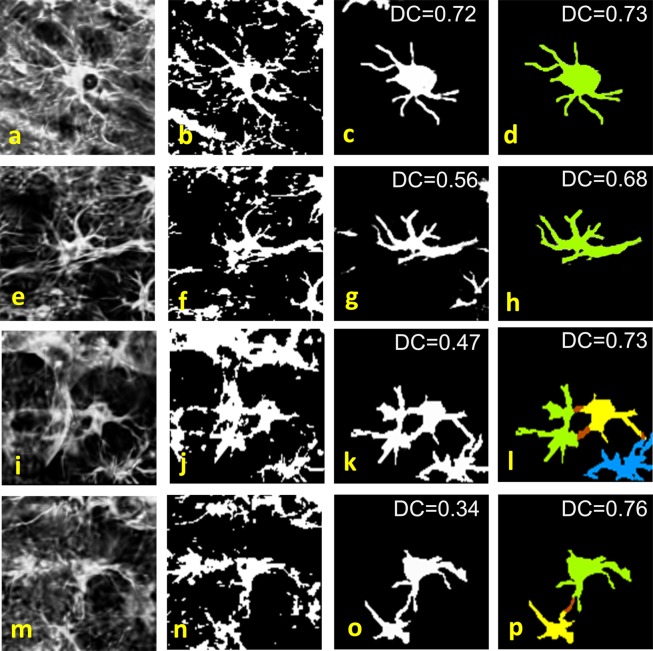
Comparison of astrocyte segmentation performance. (**a,e,i,m**) Representative image patches extracted from Fig.7(a) and containing mouse cortex astrocytes; (**b,f,j,n**) corresponding segmentation generated by the BioVoxxel algorithm in FIJI (applied locally, on the patch); (**c,g,k,o**) segmentation result using our GESU-net without post processing step; (**d,h,l,p**) segmentation result using our GESU-net including the post processing step; panels (**l,p**) use different colors (green, yellow, blue) to illustrate the separation of contiguous cells (red color indicates processes that cannot be reliably attributed to either cell). Dice’s Coefficient (DC) values are reported using GESU-net before and after postprocessing.

Since the BioVoxxel algorithm applies a global threshold on the image, its poor performance is not surprising as compared to our method that processes the astrocytes locally. Therefore, to better highlight the advantage of a learning based approach, we developed a modified version of the BioVoxxel algorithm that first applies our routine to detect the astrocytes and find an appropriate region of interest (as shown in our pipeline in Fig. 1) and next applies the BioVoxxel algorithm within each region of interest. The visual result in Fig. 7(d) shows that this approach is significantly more accurate than the direct application of the BioVoxxel algorithm to the entire image and this is reflected by the higher Dice’s Coefficient (DC = 0.65) reported in Table 2 that is now comparable to the U-net performance. We visually illustrate the difference in performance between our method and this local version of the BioVoxxel algorithm in Fig. 8 where we compare the segmentation results on selected image patches. These result show the superior ability of our approach to recover fine astrocyte processes as well as the ability of our approach to separate contiguous cells as compared with the BioVoxxel approach.

## Discussion

We have introduced a novel method for automated detection and segmentation of astrocytes in 2D microscopy images to address a challenging problem for which where very few methods are currently available.

To benchmark our detection approach, we have considered for comparison the ILASTIK software – a general purpose and user-friendy method for cell detection and classification – and FindMyCell (FMC) – a state-of-the-art method specifically designed for astrocyte detection. ILASTIK uses an approach based on machine learning that requires a training phase. However, feature selection filters available in ILASTIK are limited to conventional Gaussian and Hessian filters. As a result, while the method is able to detect bright and large cells, it is not as effective with cells exhibiting a more complex morphology like astrocytes. In the images we considered, ILASTIK tends to miss cells that appear elongated and to miss-classify large noisy regions as cells, so that its overall detection performance is rather poor (DC < 0.30). This result highlights the difficulty in applying general-purpose methods for cell analysis to astrocytes.

FMC on the other hand is designed for astrocyte detection and applies a supervised method based on deep learning. Compared with the machine learning method in ILASTIK, FMC is more flexibility and this explains its superior astrocyte detection performance. One drawback of this method is its high sensitivity to parameter selection. The FMC software requires to set two parameters: one for controlling overlapping cells and one for thresholding. We observed that even a very modest 10^−8^ change in the parameters may affect the quality of the model significantly. During the numerical experiments, we run careful tests to find the best parameters before reporting the results in Table 1. Results in the table for the image set Set_2 are consistent with the published ones^17^. As we notice above, though, the performance of the method is significantly lower on the image set Set_1 that does not belong to the Broad Bioimage Benchmark Collection on which FMC was trained.

Our method for astrocyte detection combines an unsupervised method based on Directional Ratio to detect the cells and a postprocessing routine that discards objects that do not satisfy prescribed shape criteria. Results in Table 1 show that our method significantly outperforms FMC on the image set Set_1 and its performance on Set_2 is only slightly worse than FMC. This shows that our approach performs consistently on general star-shaped cells in contrast to FMC that does not generalize well to images outside the set on which it was trained. To compare the overall detection performance between our method and FMC, we computed weighted average performance measures that reward accurate detection on image set other than the one on which the method was developed or trained. Namely, for our method we assigned a 1/3 weight when applied to Set1 and a 2/3 weight when applied to Set_2; viceversa, for FMC we assigned a 1/3 weight when applied to Set_2 and a 2/3 weight when applied to Set_1. The weighted average performance measure of the two methods are reported in Table 3 and show that our method exhibit a superior performance according to this aggregated measure.

**Table 3:** Weighted averaged cell detection performance measured using Sensitivity (S), Precision (P) and Dice’s Coefficient (DC) on our combined dataset (Set_1 and Set_2).

	S	P	DC
GESU-net	0.88	0.65	0.75
U-net	0.94	0.31	0.47
GESU-net, no post-processing	0.90	0.43	0.67
FMC	0.48	0.79	0.60

Our GESU-net algorithm is compared against a standard U-net, our GESU-net without the postprocessing module and the FindMyCell (FMC) algorithm^17^.

To assess the impact of post-processing on the detection performance of our algorithm, we included in Table 3 a comparison with our GESU-net without post-processing and a standard U-net. Results show that the postprocessing has the effect of increasing the Dice’s Coefficient of our approach from 0.67 to 0.75. The performance by the U-net is inferior to both FMC and GESU-net (with and without post-processing).

For the segmentation task, we could not compare our approach with FMC since this method does not include a segmentation routine, nor with ILASTIK since it is not designed to process star-shaped cells like astrocytes. Also we could not include a comparison with the recent work by Yang *et al*.^16^ since their methods is designed to work with 3D images and cannot be applied directly in the 2D setting.

We compared our method with the Biovoxxel algorithm that was proposed specifically for astrocyte segmentation^11^ and a standard U-net. As we observed above, the direct application of the BioVoxxel algorithm performs poorly as it misses a large number of processes emanating from the cells. We have shown that the modified BioVoxxel algorithm that works in combination with our cell detection routine and then applies the automated threshold locally performs significantly better. However, as shown in Fig. 8, it is not as accurate as our approach in segmenting finer astrocyte processes and cannot separate contiguous cells unlike our pipeline does. This highlights the advantages of using a targeted learning based strategy.

To better illustrate the advantages of our network design and the relative merit each block of our algorithmic pipeline on the segmentation performance, we reported in Table 2 a comparison of our GESU-net not only with a standard U-net but also with a modified U-net that includes a pre-trained VGG16 module, like the second block of our network. The table shows that U-net with VGG16 performs better than amstandard U-net but not as well as our GESU-net in terms of Dice’s Coefficient. Inspection of the table shows that the improvement is due to the higher Precision suggesting that GESU-net improves the True Positive count reducing potential over-segmentation.

In summary, we have shown that our multistep network-based approach for astrocyte detection and segmentation is very reliable even in images that contain dense cell populations and is very competitive against existing methods.

Even though we developed method using fluorescent images of astrocytes in the mouse cortex, we have shown that its approach is sufficiently robust to deal with astrocyte from brightfield microscopy images of astrocytes acquired from other brain regions. While it is known though astrocytes exhibit a rather diverse morphology in various brain regions, we expect that our approach would still be effective with other astrocyte populations. To ensure high segmentation performance though, it might be necessary to re-train our GESU-net including training samples representative of the populations of interest.

Finally we remark that our new pipeline offers a substrate for further quantitative analysis of astrocytes. While our network-based approach learns to segment an astrocyte, it also learns a representation of its morphology that is encoded in the network coefficients. This can be exploited to generate feature vectors according to the principles of representation learning^36,37^. Since we have shown that our pipeline has the ability to automatically detect, extract and segment astrocytes in a multicellular image with high efficiency, the same framework could be adapted to compute morphological characteristics and developed into an efficient methods for astrocyte profiling and classification.

## Data Availability

Code and imaging set Set_1 are available at https://github.com/cihanbilge/AstrocyteSegmentation. Imaging set Set_2 is available at https://data.broadinstitute.org/bbbc/image_sets.html.

## References

[1] Kettenmann, H. & Ransom, B. R. The concept of neuroglia: a historical perspective. In *Neuroglia* (Oxford University Press, 2005).

[2] Gao Y, Broussard J, Haque A, Revzin A, Lin T (2016). Functional imaging of neuron-astrocyte interactions in a compartmentalized microfluidic device. Microsystems & Nanoengineering.

[3] Clarke LE, Barres BA (2013). Emerging roles of astrocytes in neural circuit development. Nature Reviews Neuroscience.

[4] Ota, Y., Zanetti, A. T. & Hallock, R. M. The role of astrocytes in the regulation of synaptic plasticity and memory formation. *Neural plasticity* **2013** (2013). 10.1155/2013/185463 PMC3867861 24369508

[5] Verkhratsky A, Zorec R, Parpura V (2017). Stratification of astrocytes in healthy and diseased brain. Brain Pathology.

[6] Lin, C.-C. J. & Deneen, B. Astrocytes: the missing link in neurologic disease? In *Seminars in pediatric neurology*, vol. 20, 236–241 (Elsevier, 2013). 10.1016/j.spen.2013.10.004 PMC3874830 24365571

[7] Chen Y, Swanson RA (2003). Astrocytes and brain injury. Journal of Cerebral Blood Flow & Metabolism.

[8] Lacagnina MJ, Rivera PD, Bilbo SD (2017). Glial and neuroimmune mechanisms as critical modulators of drug use and abuse. Neuropsychopharmacology.

[9] Scofield MD (2016). Cocaine self-administration and extinction leads to reduced glial fibrillary acidic protein expression and morphometric features of astrocytes in the nucleus accumbens core. Biological psychiatry.

[10] Healy S, McMahon J, FitzGerald U (2018). Seeing the wood for the trees: towards improved quantification of glial cells in central nervous system tissue. Neural regeneration research.

[11] Healy S, McMahon J, Owens P, Dockery P, FitzGerald U (2018). Threshold-based segmentation of fluorescent and chromogenic images of microglia, astrocytes and oligodendrocytes in fiji. Journal of neuroscience methods.

[12] Suwannatat, P. *et al*. Interactive visualization of retinal astrocyte images. In *2011 IEEE International Symposium on Biomedical Imaging: From Nano to Macro*, 242–245 (IEEE, 2011).

[13] Ljosa, V. & Singh, A. K. Probabilistic segmentation and analysis of horizontal cells. In *Sixth International Conference on Data Mining (ICDM’06)*, 980–985 (2006).

[14] Kulkarni PM (2015). Quantitative 3-d analysis of GFAP labeled astrocytes from fluorescence confocal images. Journal of neuroscience methods.

[15] Scorcioni R, Polavaram S, Ascoli GA (2008). L-measure: a web-accessible tool for the analysis, comparison and search of digital reconstructions of neuronal morphologies. Nature protocols.

[16] Yang, L., Zhang, Y., Guldner, I. H., Zhang, S. & Chen, D. Z. 3d segmentation of glial cells using fully convolutional networks and k-terminal cut. In *International Conference on Medical Image Computing and Computer-Assisted Intervention*, 658–666 (Springer, 2016).

[17] Suleymanova, I. *et al*. A deep convolutional neural network approach for astrocyte detection. *Scientific reports* **8** (2018). 10.1038/s41598-018-31284-x PMC6110828 30150631

[18] Easley G, Labate D, Lim W-Q (2008). Sparse directional image representations using the discrete shearlet transform. Applied and Computational Harmonic Analysis.

[19] Guo K, Labate D (2007). Optimally sparse multidimensional representation using shearlets. SIAM J. Math. Anal..

[20] Guo K, Labate D, Lim W (2009). Edge analysis and identification using the continuous shearlet transform. Appl. Comput. Harmon. Anal.

[21] Labate D, Laezza F, Negi P, Ozcan B, Papadakis M (2014). Efficient processing of fluorescence images using directional multiscale representations. Math. Model. Nat. Phenom.

[22] Kayasandik, C., Guo, K. & Labate, D. Directional multiscale representations and applications in digital neuron reconstruction. *Journal of Computational and Applied Mathematics* **349** 482–493, http://www.sciencedirect.com/science/article/pii/S037704271830548X (2019).

[23] Kayasandik CB, Labate D (2016). Improved detection of soma location and morphology in fluorescence microscopy images of neurons. Journal of Neuroscience Methods.

[24] LeCun, Y., Kavukcuoglu, K. & Farabet, C. Convolutional networks and applications in vision. In *Proceedings of 2010 IEEE International Symposium on Circuits and Systems*, 253–256 (2010).

[25] Ronneberger, O., Fischer, P. & Brox, T. U-net: Convolutional networks for biomedical image segmentation. In Navab, N., Hornegger, J., Wells, W. M. & Frangi, A. F. (eds.) *Medical Image Computing and Computer-Assisted Intervention - MICCAI 2015*, 234–241 (Springer International Publishing, Cham, 2015).

[26] Atreas, N., Karantzas, N., Papadakis, M. & Stavropoulos, T. On the design of multi-dimensional compactly supported parseval framelets with directional characteristics. *Linear Algebra and its Applications* **582**, 1–36, http://www.sciencedirect.com/science/article/pii/S0024379519303155 (2019).

[27] Jacobsen, J.-H., van Gemert, J. C., Lou, Z. & Smeulders, A. W. M. Structured receptive fields in cnns. *2016 IEEE Conference on Computer Vision and Pattern Recognition (CVPR)* 2610–2619 (2016).

[28] Iglovikov, V. & Shvets, A. Ternausnet: U-net with vgg11 encoder pre-trained on imagenet for image segmentation. *arXiv preprint arXiv:1801.05746* (2018).

[29] Keras: The python deep learning library. https://keras.io/ Last accessed on 2020-01-26 (2020).

[30] Wang, J. & Perez, L. The effectiveness of data augmentation in image classification using deep learning. *Convolutional Neural Networks Vis. Recognit* (2017).

[31] Schiweck, J., Eickholt, B. J. & Murk, K. Important shapeshifter: Mechanisms allowing astrocytes to respond to the changing nervous system during development, injury and disease. *Front Cell Neurosci*. **12** (2018). 10.3389/fncel.2018.00261 PMC6111612 30186118

[32] Ru W (2019). Microglia mediate hiv-1 gp120-induced synaptic degeneration in spinal pain neural circuits. Journal of Neuroscience.

[33] Altman DG, Bland JM (1994). Diagnostic tests. 1: Sensitivity and specificity. BMJ.

[34] Ljosa V, Sokolnicki KL, Carpenter AE (2012). Annotated high-throughput microscopy image sets for validation. Nature methods.

[35] Berg, S. *et al*. ILASTIK: interactive machine learning for (bio)image analysis. *Nature Methods*, 10.1038/s41592-019-0582-9 (2019) 10.1038/s41592-019-0582-9 31570887

[36] Bengio Y, Courville A, Vincent P (2013). Representation learning: A review and new perspectives. IEEE transactions on pattern analysis and machine intelligence.

[37] Giancardo, L., Roberts, K. & Zhao, Z. Representation learning for retinal vasculature embeddings. In Cardoso, M. J. *et al*. (eds.) *Fetal, Infant and Ophthalmic Medical Image Analysis*, 243–250 (Springer International Publishing, Cham, 2017).

